# Astrocytic frataxin deficiency drives neurocognitive impairment in sickle cell mice

**DOI:** 10.1093/pnasnexus/pgag106

**Published:** 2026-04-08

**Authors:** Enrico M Novelli, Shane C Lenhart, Lesley M Foley, Nandinii Sekar, Paritosh Mondal, Hong Wang, T Kevin Hitchens, Samit Ghosh, Stephen Y Chan, Xiaoming Hu, Rimi Hazra

**Affiliations:** Pittsburgh Heart, Lung, and Blood Vascular Medicine Institute and Division of Classical Hematology, Department of Medicine, University of Pittsburgh, Pittsburgh, PA 15213, USA; Pittsburgh Heart, Lung, and Blood Vascular Medicine Institute and Division of Classical Hematology, Department of Medicine, University of Pittsburgh, Pittsburgh, PA 15213, USA; Advanced Imaging Center, Department of Neurobiology, University of Pittsburgh, Pittsburgh, PA 15203, USA; Pittsburgh Heart, Lung, and Blood Vascular Medicine Institute and Division of Classical Hematology, Department of Medicine, University of Pittsburgh, Pittsburgh, PA 15213, USA; Pittsburgh Heart, Lung, and Blood Vascular Medicine Institute and Division of Classical Hematology, Department of Medicine, University of Pittsburgh, Pittsburgh, PA 15213, USA; Department of Biostatistics, University of Pittsburgh, Pittsburgh, PA 15213, USA; Advanced Imaging Center, Department of Neurobiology, University of Pittsburgh, Pittsburgh, PA 15203, USA; Pittsburgh Heart, Lung, and Blood Vascular Medicine Institute and Division of Classical Hematology, Department of Medicine, University of Pittsburgh, Pittsburgh, PA 15213, USA; Pittsburgh Heart, Lung, and Blood Vascular Medicine Institute and Division of Classical Hematology, Department of Medicine, University of Pittsburgh, Pittsburgh, PA 15213, USA; Department of Neurology, University of Pittsburgh, Pittsburgh, PA 15213, USA; Pittsburgh Heart, Lung, and Blood Vascular Medicine Institute and Division of Classical Hematology, Department of Medicine, University of Pittsburgh, Pittsburgh, PA 15213, USA

**Keywords:** sickle cell disease, neurocognition, frataxin

## Abstract

Individuals with sickle cell disease (SCD) suffer from a high burden of neurocognitive impairment. Cerebrovascular lesions, characterized by diffusion tensor imaging identified neuroaxonal damage, are associated with learning and memory deficits. The molecular and cellular intermediates driving microstructural neuroaxonal damage and cognitive impairment in SCD remain largely unexplored. Herein, we report that sickle cell mice (SS) have reduced expression of frataxin (FXN), a mitochondrial protein, in their astrocytes compared with normal control (AA) mice. A newly generated sickle bone marrow chimeric mouse with astrocyte-specific deletion of FXN (SS^FXN-KO^) showed worsening white-matter neuroaxonal damage compared with the normal mice lacking astrocytic FXN (AA^FXN-KO^) as well as with the SS mice with wild-type FXN expression (SS^FXN-WT^). The SS^FXN-KO^ mice exhibited impaired cognitive function assessed by the functional novel object recognition (NOR) tests. Induction of FXN improved cognitive responses in the SS mice. Overall, our data demonstrate that astrocytic FXN plays a pivotal role in regulating neuroaxonal health and cognitive function in SCD.

Significance statement Cognitive deficiencies associated with white-matter injury are common among individuals with sickle cell disease (SCD), while the underlying mechanism remains unknown. In this study, we show that reduction of frataxin (FXN) in astrocytes is a major driver of white-matter damage and cognitive impairment in SCD. Reduced astrocytic FXN worsens brain injury and memory function, whereas increasing FXN improves cognitive outcomes, suggesting that targeting FXN may offer a promising therapeutic strategy to improve neurocognitive function in SCD.

## Introduction

In sickle cell disease (SCD), cerebrovascular lesions primarily consist of infarction in cortical and frontoparietal deep white-matter border zones, with resultant neuroaxonal injury (1, 2) that contributes to cognitive impairment (3–5). About 40% of children with SCD develop cerebral infarction, while about 55% of them show difficulties in learning and working memory across the lifespan (6). Cerebral infarctions are primarily diagnosed by T_2_-weighted MRI in combination with diffusion tensor imaging (DTI), which have revealed microstructural damage with reduced fractional anisotropy (FA) in SCD (7). While a proinflammatory and oxidative state in SCD may predispose individuals to neuroaxonal damage and cognitive impairment, the exact mechanism leading to cerebrovascular pathology is not known.

Astrocytes interconnect cerebral microvasculature and neurons, preserving plasticity and memory function (8). We reported that microstructural white-matter injury is associated with activation of astrocytes in sickle cell mice (SS) that simultaneously displayed poorer cognitive function compared with normal control (AA) mice (9). Frataxin (FXN) is a mitochondrial protein responsible for iron–sulfur biogenesis, and its deficiency is associated with deregulation of calcium signaling and neuroaxonal damage (10). Moreover, lack of FXN induced by hypoxia and inflammation promotes endothelial cell senescence (11), while astrocytic FXN deficiency is linked to neuronal toxicity (12). We tested whether FXN expression in the astrocytes is responsible for neuroaxonal damage and cognitive impairment in SCD.

## Results

### Astrocytic FXN is critical for neuroaxonal integrity in sickle cell mice

**Figure 1:**
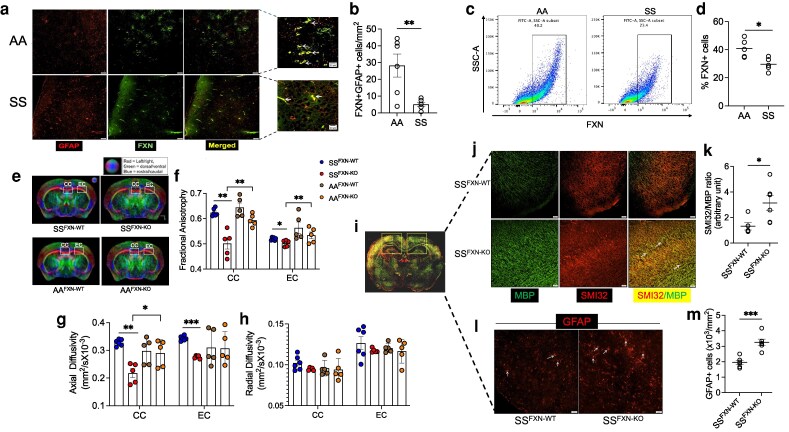
Association of FXN deficiency, neuroaxonal integrity, and cognitive impairment in sickle cell mice. a) Representative image showing decreased FXN in GFAP+ astrocytes of SS mice compared with AA mice (scale = 50 μm; inset scale bar = 20 μm). Colocalizations of FXN and GFAP are indicated by arrows in the inset images. b) Quantitation of FXN+GFAP+ cells in AA and SS brain (*n* = 6). c, d) Expression of FXN in isolated cerebral astrocytes (*n* = 5). e) Representative DEC maps for SS^FXN-WT^ and SS^FXN-KO^ mice with scalar diffusion parameters, (f) FA, (g) AD, and (h) RD for the EC and CC as indicated (*n* = 5–6). i) Representative merged stitched image of mouse brain tissue section showing combined CC and EC regions used for histopathological analysis. j, k) Immunofluorescence staining showing elevated SMI32/MBP intensity in SS^FXN-KO^ mice and SS^FXN-WT^ mice (*n* = 6; scale = 50 μm). l, m) Increased astrocyte activation marked by amplified GFAP+ staining in SS^FXN-KO^ mice (*n* = 6; scale = 50 μm).

Since reactive astrocytes accompany neurocognitive impairment and white-matter injury in SCD (9) and FXN deficiency has been linked to neuroglial dysfunction in other contexts, we investigated whether reduced astrocytic FXN contributes to neuroaxonal pathology in the SS mice. We discovered that the expression of FXN in glial fibrillary acidic protein-positive (GFAP+) reactive cerebral astrocytes of the SS mice was significantly down-regulated compared with the AA control mice (Figure 1A and B). Moreover, astrocytes isolated from whole brain tissue from AA and SS mice showed a significantly reduced number of FXN+ astrocytes in SS mice (Figure 1C and D). We generated tamoxifen-inducible astrocyte-specific FXN knockout mice (FXN-KO) by crossing FXN-floxed and *Aldh1l1*-Cre mice (Figure S1A–C). We then created bone marrow chimeric mice by transplanting whole-bone marrow cells from SS or AA mice to FXN-KO and wild-type (FXN-WT) mice (SS^FXN-KO^ and SS^FXN-WT^ or AA^FXN-KO^ and AA^FXN-WT^) followed by injection of tamoxifen to inhibit astrocytic FXN (Figure S2A–C). The chimeric mice had the hematological phenotype of AA or SS mice (Table S1). These mice brains were scanned ex vivo for MRI-DTI. Diffusion-encoded color (DEC) mapping was used to determine FA (representing microstructural damage) specifically in the corpus callosum (CC) and the external capsule (EC) areas. Although FA was modestly lower in SS^FXN-WT^ mice compared with AA^FXN-WT^ mice, it was significantly reduced only in in SS^FXN-KO^ mice (Figure 1E and F). Concurrently, the SS^FXN-KO^ mice displayed substantially reduced axial diffusivity (AD) and a slight drop in radial diffusivity (RD)—two surrogate DTI markers for neuroaxonal damage (Figure 1G and H). We analyzed the DTI data for four different genotypes within two different regions using two-way ANOVA for all four-group comparisons. For FA, significant effects of regions, genotypes, and interaction were observed. Axial diffusivity showed significant main effects of genotype and region without interaction, whereas RD demonstrated a significant effect of region only (Table S2). An increased nonphosphorylated neurofilament H (SMI32) with a concomitant decrease in myelin basic protein (MBP) indicates neuroaxonal demyelination. We quantified SMI32/MBP intensities in the CC and EC regions (Figure 1I) and found that SS^FXN-KO^ mice exhibited a marked increase in the SMI32/MBP ratio (Figure 1J and K) accompanied by a significant surge in GFAP+ astrocytes (Figure 1L and M). Although MBP immunoreactivity was increased in SS^FXN-KO^ mice, MBP intensity alone does not directly reflect functional myelin integrity. Importantly, the elevated SMI32/MBP ratio indicates increased axonal injury relative to myelin content, supporting the presence of neuroaxonal damage in SS^FXN-KO^ mice.

### Absence of FXN worsens while its induction improves neurocognition in sickle cell mice

**Figure 2:**
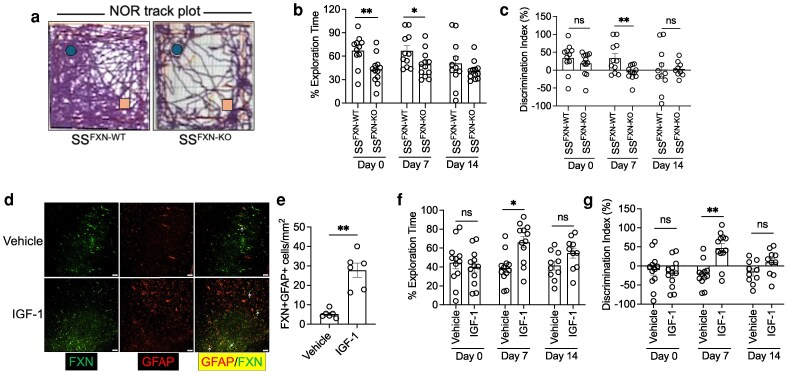
Frataxin deficiency is linked to the cognitive impairment in sickle cell mice. a) Representative track plot from NOR testing showed halted movement of SS^FXN-KO^ mice. b, c) Both SS^FXNWT^ and SS^FXN-KO^ mice were tested for NOR on weekly intervals. Reduced exploration time (b) and discrimination index (c) were evident in SS^FXN-KO^ mice (*n* = 11–12). d) The SS mice treated with recombinant IGF-1 displayed increased FXN staining in their brain tissue compared with vehicle-treated mice (scale bar = 50 μm). e) Quantitation of FXN+GFAP+ cells in IGF-1 and vehicle-treated mice (*n* = 6). f, g) The SS mice treated with IGF-1 showed improved cognitive function in NOR testing compared with vehicle-treated mice (*n* = 11–12). ns, nonsignificant; *P < 0.05, **P < 0.01 (unpaired t test).

Since neuroaxonal damage is associated with impaired cognitive function in both sickle cell mice and sickle patients (9, 13), we determined whether loss of astrocytic FXN leads to poor cognitive function in SS mice. We performed a novel object recognition (NOR) test for determining behavioral learning and working memory in mice. The SS^FXN-KO^ mice had continuity gaps in their movement and spent significantly less time exploring the novel object with inefficiency to differentiate the familiar object and the novel object compared with the SS^FXN-WT^ mice. Interestingly, while SS^FXN-KO^ mice exhibited reduced object exploration compared with SS^FXN-WT^ mice (SS^FXN-KO^: 62.94 ± 10.29 s vs. SS^FXN-WT^: 106.6 ± 12.35 s), total exploration time was comparable, indicating that the observed reduction in novel object preference was due to impaired cognitive performance rather than generalized hypoactivity (Figure 2A–C). Insulin growth factor-1 (IGF-1) nonspecifically stimulates FXN (14). Prophylaxis with human recombinant IGF-1 considerably induced astrocytic FXN in SS mice (Figure 2D and E) and augmented exploration time and discrimination index in the SS mice compared with the vehicle-treated ones (Figure 2F and G). One-way ANOVA across different time points (day 0, day 7, and day 14) of NOR measurements revealed no significant effect of time on percent exploration time or discrimination index in either the SS^FXN-WT^ vs. SS^FXN-KO^ or the vehicle vs IGF-1 cohort, indicating that multiple NOR tests across time did not account for the observed group differences (Table S3).

## Discussion

In this study, we have demonstrated a direct correlation between astrocytic FXN and neuroaxonal injury with cognitive outcomes in a preclinical model of SCD. The DTI analysis revealed substantial microstructural damage exclusively in SS^FXN-KO^ compared with both SS^FXN-WT^ and AA^FXN-KO^ mice. This suggests that astrocytic FXN deficiency–associated SCD genotype is linked to the microstructural damage in CC and EC areas of the brain. Intact DTI features in AA^FXN-WT^ and AA^FXN-KO^ mice indicate radiation during bone marrow transplantation had minimal effect on neuroaxonal integrity. Intrinsically reduced IGF-1 is associated with white-matter injury in SCD (15). In accordance with its potential neuroprotective role, our findings demonstrate improved cognitive responses with exogenous IGF-1 in conjunction with increased FXN expression. Because IGF-1 has pleiotropic actions, cognitive improvement may involve both FXN-dependent and FXN-independent pathways, precluding clear mechanistic attribution. While the IGF-1 treatment provides a proof of concept, future studies employing FXN-targeted approaches will help refine FXN-selective therapeutic strategies for enhancing neurocognitive health in SCD.

## Materials and methods

Refer to Supplementary material for detailed methodology.

### Animals

The knock in Townes’ sickle cell mice homozygous for human Hb^S^ (SS) or human Hb^A^ (AA) (#013071), floxed FXN mice (FXN^fl/fl^ #028520) and Aldh1l1-Cre/ERT2 BAC (#031008) from The Jackson Laboratories were used. All experimental protocols followed Institutional Animal Care and Use Committee approval (#22010095 and #24126048). All experiments included male and female AA and SS mice (12–14 weeks).

### Bone marrow transplantation

The newly generated FXN^KO^ mice lacking astrocytic FXN and FXN^fl/fl^ mice expressing FXN (referred to herein as FXN^WT^) were transplanted with whole-bone marrow cells from AA and SS mice to generate chimeric mouse strains.

### Diffusion tensor imaging

Ex vivo DTI was performed using a Bruker AV3HD 11.7 Tesla/89 mm vertical bore microimaging system and ParaVision 6.0.1 (Bruker BioSpin, Billerica, MA, United States). DTI data were quantified using DSI Studio to determine mean FA, AD, and RD.

### Immunofluorescence

The paraformaldehyde-fixed, paraffin-embedded parietal brain tissue sections (5 μm) were assessed for FXN, SMI32, MBP, and GFAP. Images were captured with an Olympus APX100 microscope and analyzed by CellSensAPEX and Fiji-Image J software.

### Isolation of cerebral astrocytes

Mouse primary astrocytes were isolated using microbeads labeled with the astrocyte-specific antibody for astrocyte cell surface antigen (anti-ACSA-2 antibody).

### In vivo cognitive assessment

The learning and memory function of the mice was assessed using NOR tests. The time spent exploring a familiar and novel object was analyzed using Anymaze software.

### Statistical analysis

Normally distributed data were analyzed using a two-tailed unpaired Student's t test. One- or two-way ANOVA tests were performed wherever appropriate. GraphPad Prism 10 software was used for generating the graphs and performing all statistical analyses.

## Supplementary Material

pgag106_Supplementary_Data

## Data Availability

All data are included in the manuscript and/or supporting information.

## Supplementary Material

Supplementary materials including detailed methodology and supplementary data are available at *PNAS Nexus* online.

## Preprints

This manuscript was posted on a preprint at https://www.biorxiv.org/content/10.1101/2024.12.20.629760v1.
