# Supplementary material for: Astrocytic frataxin deficiency drives neurocognitive impairment in sickle cell mice
Source: PNAS Nexus. 2026 Apr 8;5(4):pgag106. doi: 10.1093/pnasnexus/pgag106 (PMC13108596; doi:10.1093/pnasnexus/pgag106)
Supplement: pgag106_Supplementary_Data [file pgag106_supplementary_data.pdf]

# Supporting Information (Supplemental Figures and Detailed Methods)

## Astrocytic frataxin deficiency drives neurocognitive impairment in sickle cell mice

Enrico M. Novelli[1], Shane C Lenhart[1], Lesley M. Foley[2], Nandinii Sekar[1], Paritosh Mondal[1], Hong Wang[3], T. Kevin Hitchens[2,4], Samit Ghosh[1], Stephen Y. Chan[1], Xiaoming Hu[5] and Rimi Hazra[1*]

[1]Pittsburgh Heart, Lung, and Blood Vascular Medicine Institute and Division of Classical Hematology, Department of Medicine, [2]Advanced Imaging Center, [3]Department of Biostatistics, [4]Department of Neurobiology, [5]Department of Neurology, University of Pittsburgh, Pittsburgh, PA, USA

*Corresponding author:
Rimi Hazra
rih17@pitt.edu

### Supplementary Figures

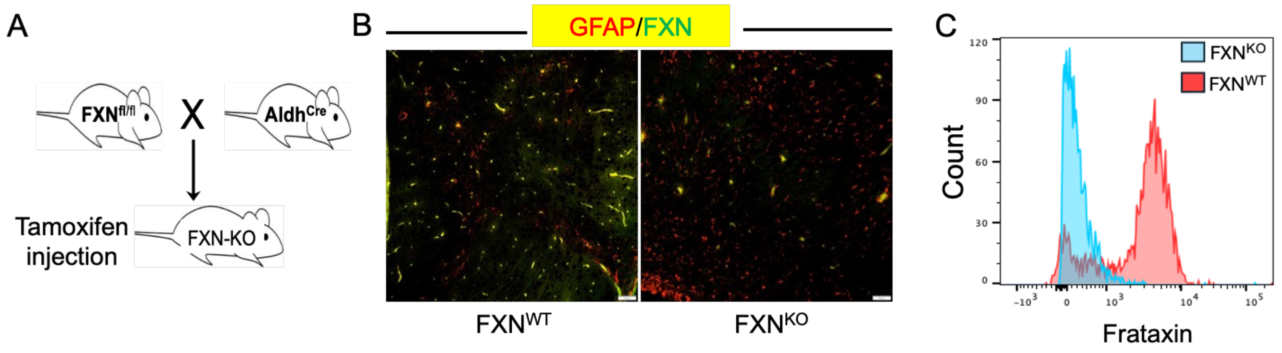


**Supplementary Figure 1. Generation of mouse strain with targeted deletion of frataxin in astrocytes. (A)** General breeding schema between FXN$^{fl/fl}$ and Aldh$^{Cre}$ mice. **(B)** Representative cerebral tissue sections from FXN$^{WT}$ and FXN$^{KO}$ mice showing coexpression of FXN and GFAP (astrocyte marker) (scale bar=50 μm). **(C)** Flow analysis showing lack of FXN expression in the isolated astrocytes from the indicated group of mice.

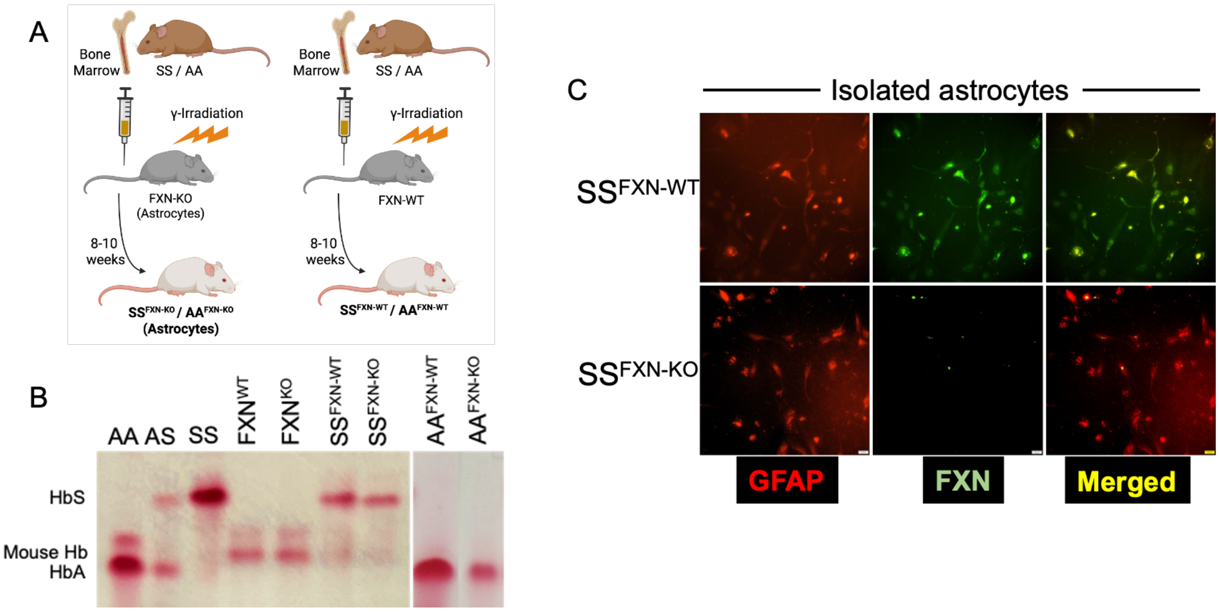


**Supplementary Figure 2. Bone marrow transplantation and astrocyte specific FXN expression in SS$^{FXN-KO}$ and SS$^{FXN-WT}$. (A)** Schema showing generation of bone marrow chimera mice. **(B)** Hemoglobin (Hb) gel electrophoresis showing expression of normal (HbA) and sickle Hb (HbS) in chimera mice as indicated. **(C)** Representative immunofluorescence images showing expression of FXN in GFAP+ astrocytes in SS$^{FXN-KO}$ and SS$^{FXN-WT}$ mice.

| | AA | SS | FXN$^{-WT}$ | FXN$^{-KO}$ | SS$^{FXN-KO}$ | SS$^{FXN-WT}$ | AA$^{FXN-KO}$ | AA$^{FXN-WT}$ |
|---|---|---|---|---|---|---|---|---|
| Hb (g/dl) | 11.8 ± 1.9 | 6.9 ± 1.6 | 13.4 ± 0.33 | 12.1 ± 0.13 | 9.2 ± 0.51 | 8.3 ± 0.51 | 11.8 ± 0.9 | 12.5 ± 1.39 |
| HCT (%) | 41.3 ± 1.7 | 30.3 ± 2.9 | 42.37 ± 1.8 | 43.21 ± 1.9 | 34.61 ± 2.39 | 33.51 ± 1.80 | 42.51 ± 3.9 | 43.7 ± 2.8 |
| Retics (%) | 6.5 ± 1.1 | 59.6 ± 5.2 | 7.89 ± 1.06 | 10.29 ± 3.1 | 51.7 ± 7.19 | 43.88 ± 5.53 | 5.29 ± 1.3 | 6.91 ± 1.26 |
| WBC ($10^3$/µl) | 7.4 ± 1.1 | 21.2 ± 8.1 | 6.18 ± 1.18 | 8.37 ± 0.94 | 19.6 ± 2.98 | 27.5 ± 3.84 | 6.71 ± 1.4 | 6.73 ± 1.21 |
| RBC ($10^6$/µl) | 5.7 ± 1.3 | 6.2 ± 2.13 | 8.71 ± 1.62 | 7.27 ± 1.38 | 7.02 ± 2.18 | 6.36 ± 1.08 | 6.7 ± 1.81 | 7.18 ± 1.92 |
| Platelet ($10^3$/µl) | 368.6 ± 27.2 | 398.2 ± 47.2 | 499.7 ± 98.1 | 378.2 ± 28.1 | 451.7 ± 71.2 | 415.6 ± 90.6 | 418.2 ± 23.3 | 487.2 ± 43.1 |

**Supplementary Table 1.** Hematological characteristics donor, recipient and bone marrow chimera mice. Data represented as mean ± SEM.

| Parameters | Effect | F (df1, df2) | P value |
|---|---|---|---|
| **Fractional Anisotropy (FA)** | Interaction | 5.44 (3,34) | 0.0036 |
| | Region | 39.08 (1,34) | <0.0001 |
| | Genotype | 18.41 (3,34) | <0.0001 |
| **Axial Diffusivity (AD)** | Interaction | 0.76 (3,34) | 0.5231 |
| | Region | 4.40 (1,34) | 0.0434 |
| | Genotype | 8.99 (3,34) | 0.0002 |
| **Radial Diffusivity (RD)** | Interaction | 0.03 (3,34) | 0.9938 |
| | Region | 46.03 (1,34) | <0.0001 |
| | Genotype | 1.49 (3,34) | 0.2340 |

**Supplementary Table 2.** Two-way ANOVA (Genotype x Region) for DTI measures

| | Parameter | F (df1, df2) | P value | Significant |
|---|---|---|---|---|
| **$SS^{FXN\text{-}WT}$ vs $SS^{FXN\text{-}KO}$ (NOR)** | % Exploration Time | 0.4862 (2,3) | 0.6563 | No |
| | Discrimination Index | 0.9687 (2,3) | 0.4736 | No |
| **Vehicle vs IGF-1 (NOR)** | % Exploration Time | 0.3346 (2,3) | 0.7393 | No |
| | Discrimination Index | 0.4149 (2,3) | 0.6933 | No |

**Supplementray Table 3.** One-way ANOVA across time points (Day 0, Day 7 and Day 14)

**Supplementary Methods**

**Sex as a biological variable**

Both male and female mice were used, and similar findings are reported for both sexes.

***Mice and Bone marrow transplantation***

The University of Pittsburgh Institutional Animal Care and Use Committee (22010095 and 24126048) approved all mouse studies. The AA and SS mice were 12-14 weeks old at the time of the experiments. Knock-in Townes' SCD mice (#013071) (1) expressing human-Hb $\beta^S$ (SS) and control mice with human-Hb $\beta^A$ (AA) were purchased from Jackson Laboratories and bred in the University of Pittsburgh vivarium. Mouse genotypes were confirmed either by PCR or Hb gel electrophoresis. We bred floxed frataxin (FXN) mice ($FXN^{fl/fl}$; C57BL/6J-*Fxn*$^{em2Lutzy}$/J; #028520) with Aldh1l1-Cre/ERT2 BAC mice (B6N.FVB-Tg(Aldh1l1-cre/ERT2)1Khakh/J; #031008) to generate tamoxifen-inducible deletion of FXN specifically in *Aldh1l1*-expressing astrocytes ($FXN^{KO}$). The $FXN^{fl/fl}$ mice (referred herein as $FXN^{WT}$) (6-8 weeks of age, both sexes) were maintained on acidified drinking water for 7 days and subjected to one dose of 1200 rads irradiation. Irradiated mice were transplanted with $5x10^6$ whole bone marrow cells harvested from SS mouse donors. The transplanted mice (designated as $SS^{FXN\text{-}KO}$, $SS^{FXN\text{-}WT}$, $AA^{FXN\text{-}KO}$ and $AA^{FXN\text{-}WT}$) were maintained on medicated (Neomycin: 0.5mg/ml; Polymyxin B: 0.0125 mg/ml) water for one week. Mice were phlebotomized by retro orbital bleeding using a capillary tube internally coated with heparin/EDTA anticoagulant. Blood samples were collected from transplanted mice 8-weeks post-transplant for complete blood count (CBC) using HemaTrue hematology analyzer (Heska). To assess reticulocyte count using flow cytometry. Percent reticulocytes were determined by flow cytometric analysis of thiazole orange stained whole blood samples (2) and analyzing data using a FACSDIVA software. Hb-gel electrophoresis was performed to identify the presence of HbS on the transplanted sickle bone marrow chimera mice.

***Tamoxifen induction:*** . In brief, tamoxifen was dissolved in corn oil at 20 mg/ml at 37°C overnight, diluted to 15 mg/ml. It was administered intraperitoneally at 100 µl per mouse for five consecutive days. **i)** Tamoxifen was initially injected in newly generated $FXN^{KO}$ to confirm astrocytic FXN deletion. **ii)** Following recipient bone marrow engraftment, tamoxifen was injected in $AA^{FXN\text{-}KO}$ and $SS^{FXN\text{-}KO}$ mice to suppress the astrocytic FXN expression in the sickle bone marrow chimera mice. The MRI, flow cytometry or NOR experiment were initiated 7-day following the last tamoxifen injection.

***Insulin growth factor-1 Treatment***

Recombinant human IGF-1 (50 µg/kg, ab9573) or vehicle (phosphate buffer saline, PBS) were injected subcutaneously for 5 days.

***Diffusion tensor imaging (DTI)***

Ex vivo diffusion tensor imaging (DTI) was performed using a Bruker AV3HD 11.7 Tesla/89 mm vertical-bore microimaging system equipped with a Micro2.5 gradient set, a 20 mm quadrature radiofrequency resonator, and ParaVision 6.0.1 software (Bruker BioSpin, Billerica, MA). DTI data were acquired using a multislice spin-echo sequence with five non–diffusion-weighted (b0) images and 30 non-collinear diffusion-weighted directions. Imaging parameters were as follows: echo time/repetition time (TE/TR) = 22/2800 ms, two signal averages, matrix size = 160 × 160, field of view = 16 × 16 mm, 25 slices, slice thickness = 0.5 mm, b-value = 3000 s/mm², and diffusion gradient timing $\Delta/\delta$ = 11.0/5.0 ms. DTI datasets were processed and analyzed using DSI Studio (http://dsi-studio.labsolver.org/)(3). Regions of interest (ROIs) were manually delineated in the corpus callosum (CC) and external capsule (EC) of the left and right hemispheres to extract mean fractional anisotropy (FA), axial diffusivity (AD), and radial diffusivity (RD)

***Immunofluorescence***

Following DTI acquisition, whole brains were harvested and sectioned into 3 mm coronal slices. The second slice from the anterior half of each brain was processed for paraformaldehyde-fixed, paraffin-embedded sectioning (5 µm). Six sections per mouse were obtained from this slice for immunofluorescence analysis. Brain sections from AA, SS, SSFXN-WT, and SSFXN-KO mice were assessed for white matter injury using double immunofluorescence staining for non-phosphorylated neurofilament H (SMI32; BioLegend, cat. #801702) and myelin basic protein (MBP; Proteintech, cat. #10458-1-AP). Additional immunostaining was performed to detect axonal injury, astrocyte activation using glial fibrillary acidic protein (GFAP; Proteintech, cat. #16825-1-AP), and frataxin (FXN; Abcam, cat. #ab197963). Images were acquired using an Olympus APX 100 microscope. Quantification of MBP and SMI32 staining was performed within the corpus callosum and external capsule using Fiji (ImageJ). Mean staining intensities from six sections were averaged to calculate the SMI32/MBP ratio for each mouse.

***Isolation of cerebral astrocytes and flow cytometry***

Primary astrocytes were isolated from single-cell suspensions of mouse brain tissue using microbeads labeled with the astrocyte-specific anti-ACSA-2 antibody (Miltenyi Biotec; #130-095-826). Isolated astrocytes were washed with cold buffer and centrifuged at 300 × g for 5 min at 4°C, then fixed and permeabilized for 30 min at 4°C in the dark. Following two washes with cold permeabilization buffer, cells were incubated with FXN antibody for 30 min at 4°C in the dark, washed again, and resuspended in cold buffer for acquisition. Flow cytometry was performed on a Fortessa cytometer, and the expression of FXN in astrocytes were analyzed using FlowJo.

### ***In vivo cognitive assessment (4)***

The SS, $SS^{FXN\text{-}WT}$ and $SS^{FXN\text{-}KO}$ mice were used to perform the Novel Object Recognition (NOR) Test to evaluate learning and memory deficits. One day following the last tamoxifen injection in $SS^{FXN\text{-}KO}$ mice, all mice were placed in the center of the arena (a plastic chamber measuring 40 cm long × 40 cm wide × 35 cm high) for 10 minutes to gain familiarity with two identical objects placed 25 cm away in two corners (~8 cm from adjacent corner walls). Following the pre-test phase, mice were placed in the home cage for one hour. For the novel object test phase, one original object ( ) was replaced by a novel object ( ). Both objects were consistent in height and volume but different in shape and color), and mice were placed back into the arena for 3 minutes. Object exploration was recorded when the mice approached an object with their snout within a 2-cm perimeter around the object; the time spent exploring both objects was recorded. The exploration time (%) [(exploration time with novel object / total exploration time) × 100] and the discrimination index [(exploration time with novel object − exploration time with familiar object)/ total exploration time] were calculated (5). One day prior to NOR data collection, the mice were pre-conditioned. Data were collected 3 times marked herein as Day 0, Day 7, and Day 14.

### ***Statistical analysis***

Data are presented as mean ± SEM with individual data points shown. Normality was assessed using the Shapiro–Wilk test and visual inspection of histograms. Statistical comparisons between groups were performed using two-tailed unpaired Student's *t*-tests. One-way (to compare three time points of NOR measurement) or Two-way ANOVA (to compare four different mouse strains and two different regions) were performed. All analyses and graphical presentations were generated using GraphPad Prism 10 (GraphPad Software). P-values are reported in the figure legends, and statistical significance was defined as $p<0.05$.

## **References to the Supplementary materials**


1. L. C. Wu *et al.*, Correction of sickle cell disease by homologous recombination in embryonic stem cells. *Blood* **108**, 1183-1188 (2006).
2. P. R. Nobes, A. B. Carter, Reticulocyte counting using flow cytometry. *J Clin Pathol* **43**, 675-678 (1990).
3. F. C. Yeh, T. D. Verstynen, Y. Wang, J. C. Fernandez-Miranda, W. Y. Tseng, Deterministic diffusion fiber tracking improved by quantitative anisotropy. *PLoS One* **8**, e80713 (2013).
4. R. Hazra *et al.*, White matter abnormalities and cognitive dysfunction are linked to astrocyte activation in sickle mice. *PNAS Nexus* 10.1093/pnasnexus/pgad149 (2023).

5. H. Pu *et al.*, Intranasal delivery of interleukin-4 attenuates chronic cognitive deficits via beneficial microglial responses in experimental traumatic brain injury. *J Cereb Blood Flow Metab* **41**, 2870-2886 (2021).
